# Multilayer Composite Structured Transparent Infrared-Selective Stealth Films with Synergistic Radiative Cooling

**DOI:** 10.3390/nano16161038

**Published:** 2026-08-20

**Authors:** Juantao Zhang, Haining Ji, Shisong Jin, Zhiwen Wu, Yuzhuo Ma, Jianfeng Li, Guanhong Lu, Chang Cheng, Xiangle Li

**Affiliations:** 1 School of Physics and Optoelectronics, Xiangtan University, Xiangtan 411105, China; 2 Hunan Engineering Laboratory for Microelectronics, Optoelectronics and System on a Chip, Xiangtan University, Xiangtan 411105, China

**Keywords:** multilayer films, transparent, infrared-selective stealth, radiative cooling

## Abstract

Infrared-selective stealth films, which concurrently offer high visible transmittance, suppressed infrared emission, and selective thermal dissipation, have emerged as compelling candidates for infrared protection and thermal-target stealth. However, traditional multilayer architectures are predominantly designed through empirical trial-and-error protocols, which inherently hinder the synergistic optimisation of multiband spectral performance and yield suboptimal parameter-tuning efficiency. To circumvent this bottleneck, we introduce a reinforcement learning (RL)-driven multi-objective optimisation framework that automates the design of composite thin-film configurations. The optimised multilayer film structure consists of TiO_2_/ITO/Ag/ZnO/SiO_2_, with layer thicknesses of 180, 656, 10, 33.75 and 50 nm, respectively. Spectral characterisation reveals a weighted average visible transmittance of 79.77% over the 0.38–0.78 μm range, alongside blackbody-weighted average emissivities of 33.93%, 72.93%, and 19.94% in the 3–5, 5–8, and 8–14 μm bands, respectively. Consequently, the spectral profile exhibits high visible transparency, deep suppression of emissivity within the atmospheric windows (3–5 and 8–14 μm), and markedly elevated emissivity in the non-atmospheric band (5–8 μm). Analysis of the electromagnetic field distribution and power-loss density along the thickness direction reveals that the energy transmission and dissipation behaviours across distinct bands are synergistically governed by multilayer interference, interfacial multiple reflections, and lossy interlayer coupling mechanisms. Furthermore, angle-resolved infrared-emissivity analysis calibrated against the normal-incidence FDTD spectrum confirms that the structure retains robust polarisation adaptability and pronounced spectral selectivity at incidence angles up to 80°. The above results demonstrate the effectiveness of the reinforcement learning-driven optimisation framework for the automated co-design of multiband spectral responses. Moreover, the uncovered multilayer interference and loss-coupling mechanisms furnish a solid physical foundation for further performance refinement and rational design of transparent stealth coatings.

## 1. Introduction

**Figure 1:**
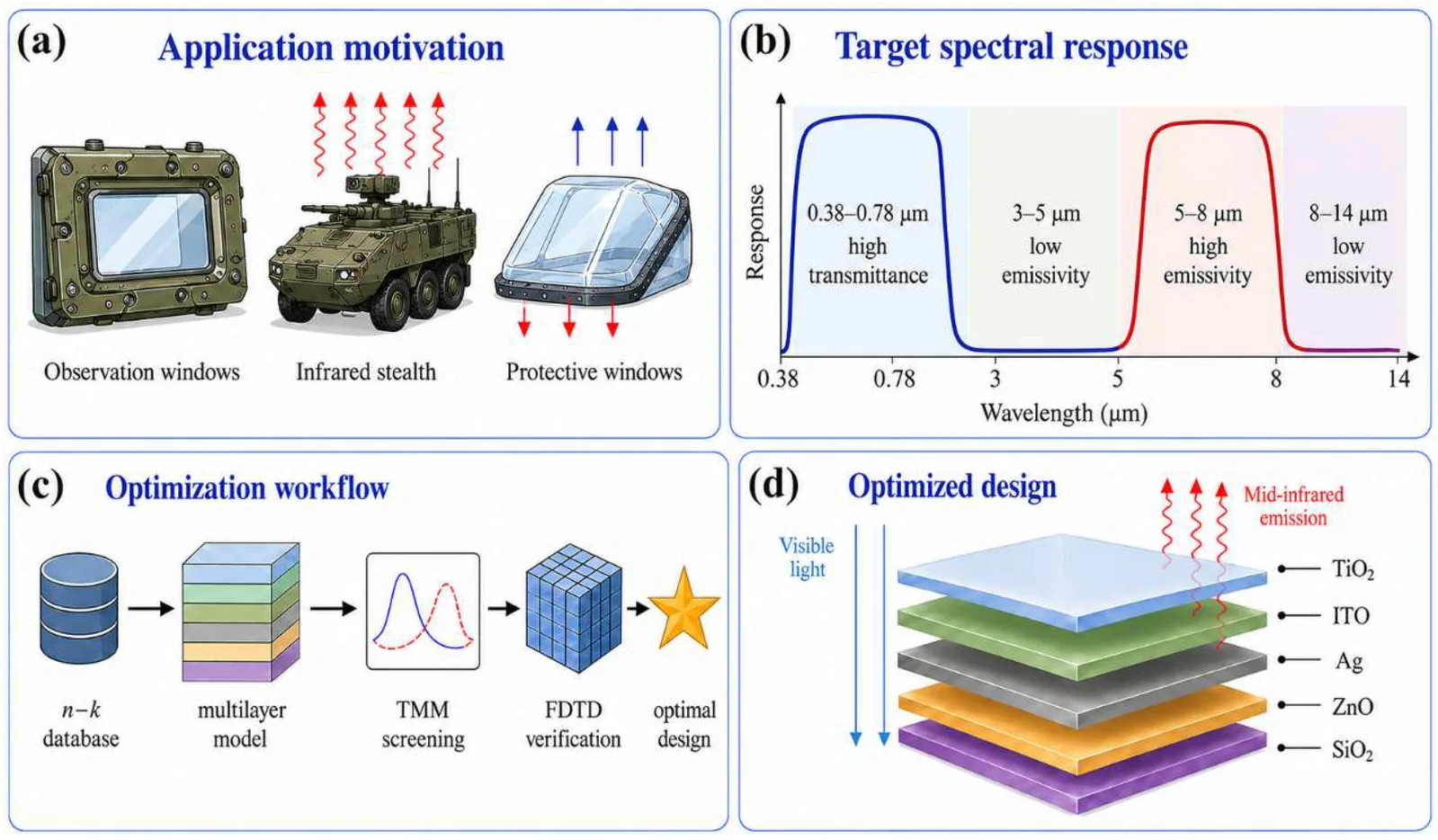
(**a**) Schematic diagram of the application scenario; (**b**) schematic diagram of the target spectral response; (**c**) schematic diagram of the multilayer film optimisation process; (**d**) schematic diagram of the optimised structure and spectral tuning.

The concurrent advancement of visible and infrared detection technologies has rendered single-band stealth insufficient for safeguarding equipment in complex battlefield environments, thus driving considerable research interest in visible–infrared-compatible stealth materials [1,2,3,4]. As shown in Figure 1a, such materials can be used in transparent components such as armoured vehicle observation windows, aircraft canopies and protective windows for electro-optical equipment, enabling the underlying colour, texture and camouflage information to be effectively displayed in the visible-light spectrum, while simultaneously reducing the target’s radiative signature within the infrared atmospheric window [5,6,7]. However, the spectral requirements across different wavebands are inherently conflicting: strong visible absorption or reflection by the outer layer would obscure the camouflage beneath, whereas a purely broadband low-emissivity approach in the infrared would impede heat release, thereby raising surface temperature and undermining sustained stealth performance [8,9,10]. Consequently, the outer transparent functional film must not only exhibit high visible transmittance but also suppress infrared emission within the atmospheric windows and promote selective thermal radiation outside these bands. Its spectral control properties determine not only the rendering of visible-light information from the underlying layers but also influence the target’s infrared radiation characteristics, operational temperature rise and long-term stealth stability [11,12,13]. Consequently, a synergistic optimisation of visible transparency, infrared stealth, and selective heat dissipation within the outer-layer structure is imperative.

Achieving the multifunctional compatibility described above hinges on establishing a selective spectral response that meets the specific requirements of different wavelength bands. As shown in Figure 1b, an ideal transparent infrared stealth material must maintain high transmittance in the 0.38–0.78 μm visible-light band, low emissivity within the two atmospheric windows of 3–5 μm, mid-infrared, and 8–14 μm, long-wave infrared, and high emissivity in the 5–8 μm non-atmospheric window to enable efficient thermal dissipation [14,15,16,17,18]. However, these spectral requirements are inherently contradictory: increasing visible transparency generally demands minimising absorption and interfacial reflection, while reducing mid- and long-wave infrared emissivity requires enhancing the structure’s reflectivity in those bands. Moreover, conventional broadband low-emissivity materials, though effective at attenuating infrared signals, also impede the outward release of internally generated heat, causing both the internal and surface temperatures to rise progressively and thereby compromising long-term stealth performance. Consequently, how to redirect thermal radiation energy from the 3–5 μm and 8–14 μm detection windows to the 5–8 μm non-atmospheric window while maintaining visible transparency has become a central issue in research [19,20,21,22].

Although existing transparent conductive films, metal-dielectric composites, and multilayer interference structures have demonstrated certain advances [23,24,25,26], they still suffer from inherent trade-offs due to material optical constants, interfacial coupling, and thickness constraints. Specifically, poor balance between visible transparency and low infrared emissivity, insufficient spectral selectivity, and degraded performance at oblique incidence. Multilayer films, by exploiting interference and impedance modulation among transparent conductive oxides, ultrathin metals, and dielectrics of different refractive indices, offer a promising route to achieving the desired spectral profile: high visible transmittance, suppressed emission within atmospheric windows, and enhanced emission outside them. However, their spectral response is governed by a complex interplay of refractive indices, layer sequence, thickness, and incidence angle, resulting in a high-dimensional parameter space even for a few functional layers. Conventional trial-and-error optimisation, relying on empirical layer-by-layer tuning, is inefficient and often fails to capture the nonlinear coupling among layers, making it difficult to strike a stable balance among conflicting performance criteria [27,28,29]. Genetic algorithms, particle swarm optimisation, and supervised-learning-based methods have been increasingly applied to the optimisation of complex photonic structures, improving the efficiency of parameter exploration and inverse design [30,31,32,33]. Reinforcement learning further provides a sequential decision-making framework in which an agent continuously interacts with the physical environment and updates structural parameters according to multiband spectral-performance feedback. This closed-loop strategy offers an effective approach for the automated collaborative optimisation of finite-dimensional multilayer films with coupled spectral objectives [34,35].

Herein, we propose a reinforcement learning-driven multi-objective optimisation framework for the simultaneous selection of materials and geometric parameters in transparent infrared-selective composite films, aiming at a spectral response that combines high visible transmittance, low emissivity within the atmospheric windows, and enhanced emissivity in the non-atmospheric window. Full-wave simulations, power-loss analysis, and angular-resolved calculations are then used to elucidate the underlying spectral control mechanisms, thereby providing practical guidelines for the design and performance tuning of such stealth films.

## 2. Optimisation Methodology

**Figure 2:**
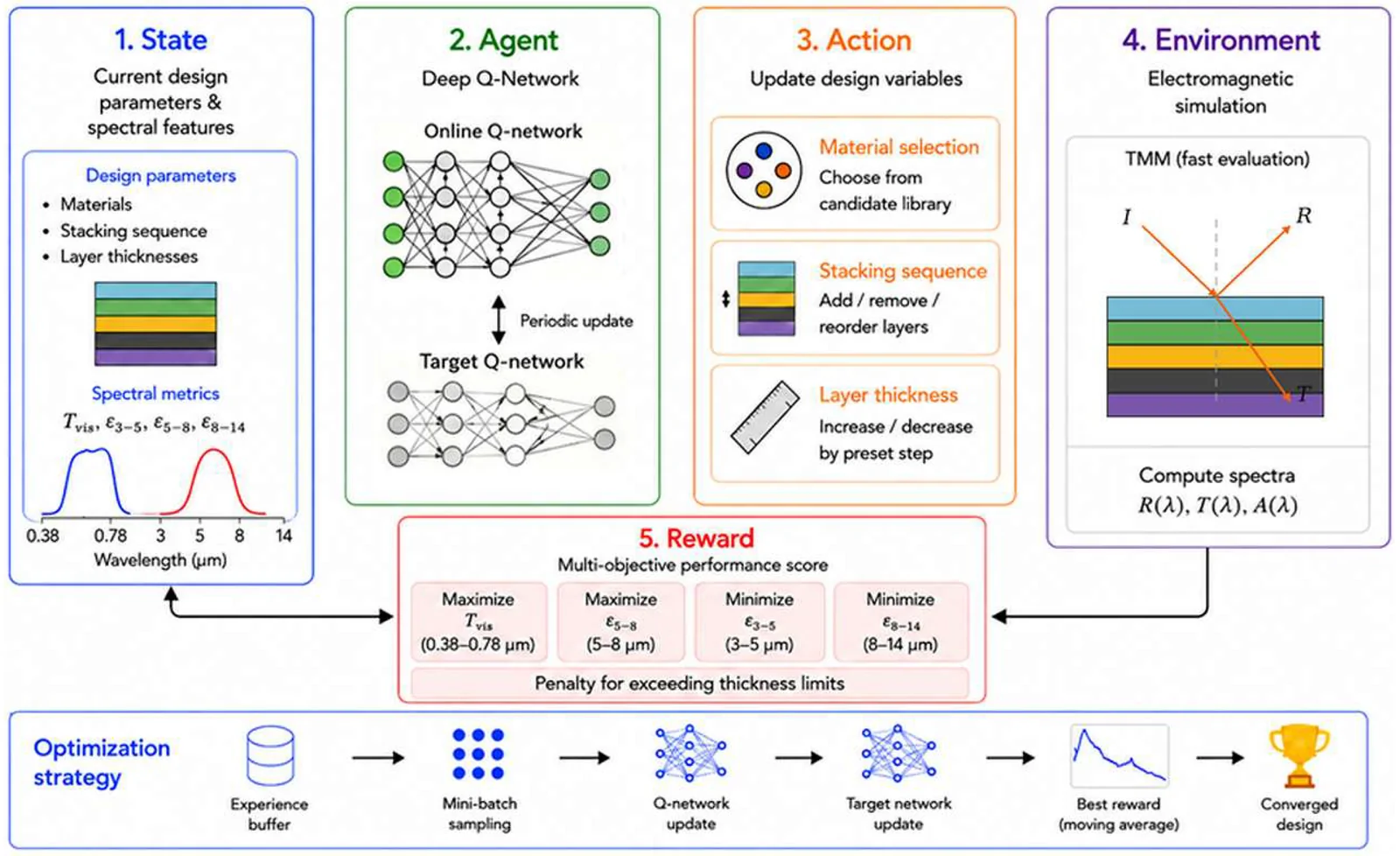
Reinforcement learning-driven optimisation workflow, showing the main interaction loop and DQN training procedure.

This study presents a reinforcement learning-assisted multi-objective optimisation framework for transparent infrared-selective stealth films, where material choices, stacking sequence, and thickness parameters are jointly screened within a predefined candidate-material and structural-parameter space. The transfer-matrix method (TMM) serves as a rapid spectral-evaluation environment [36], and iterative interaction between the agent and the optical model achieves coordinated optimisation of visible transparency, infrared-emission suppression, and selective radiative heat dissipation. The overall workflow is shown in Figure 2. As shown in Figure 2, the current design parameters and corresponding spectral features constitute the state representation of the agent, while the action space includes updates to material selection, layer number, stacking sequence, and layer thickness within the predefined design space. After the structure is updated, the TMM rapidly calculates the reflectance, transmittance, and absorptance of the candidate design, from which a multiband reward is obtained. The updated structural parameters, spectral response, and reward are then returned to the agent, forming a closed optimisation loop of state–action–spectral evaluation–reward–state update.

An ε-greedy policy is adopted to balance exploration and exploitation. Each transition (state, action, reward, next state) is stored in an experience replay buffer, from which mini-batches are randomly sampled to update the online Q-network; the target network is periodically synchronised to mitigate training instability. The optimisation process is terminated when the moving-average reward stabilises and the best overall performance shows no further improvement over several consecutive episodes.

### 2.1. Structural Parametrisation and Material Selection

To establish a design space for transparent, infrared-selective composite films, this paper selects candidate materials capable of visible-light transmission, infrared reflection, spectral absorption, and interface matching and implements a standardised coding system for film material categories, arrangement sequences, and thickness parameters. The candidate materials encompass functional types such as high-refractive-index dielectrics, transparent conductive oxides, ultrathin metals and low-loss dielectrics: high-refractive-index dielectrics are primarily used to modulate the interference phase within the film; transparent conductive oxides are used to balance visible-light transmission with infrared optical response; ultrathin metals are used to enhance mid- and far-infrared reflection; and low-loss dielectrics are used to improve interface matching and light field distribution. Reinforcement learning was employed to conduct a joint screening within the predefined material library and thickness constraints, ultimately yielding a five-layer composite structure comprising TiO_2_/ITO/Ag/ZnO/SiO_2_.

For each set of candidate structures, the reflectance R(λ) and transmittance T(λ) are first calculated; the absorptance is then derived from the law of conservation of energy:

(1) A(λ)=1−R(λ)−T(λ)

Under conditions of thermal equilibrium, according to Kirchhoff’s law, the spectral emissivity of a structure can be characterised by its absorptivity at the same wavelength and in the same direction [37].

### 2.2. Reinforcement Learning-Based Multi-Objective Optimisation

This paper employs a discrete-action reinforcement learning policy based on a deep Q-network. The agent’s state comprises the current structural parameters and four key spectral-band metrics, while the action space corresponds to updating the material selection, layer number, stacking sequence, or layer thickness within the predefined design space. The online Q-network estimates the expected long-term return associated with each action under the current state. The TMM rapidly evaluates the spectral response of the updated structure, and the resulting multi-objective performance is returned to the reward module, thereby forming the closed optimisation loop shown in Figure 2.

The reward function is primarily designed to achieve high transmittance of visible light in the 0.38–0.78 μm range, emission suppression in the 3–5 μm and 8–14 μm ranges, and high emission in the 5–8 μm range. In this framework, visible-light transmittance and the 5–8 μm emissivity serve as positive rewards, while the 3–5 μm and 8–14 μm emissivities act as penalty terms. Additionally, a penalty for exceeding thickness limits and a reward for optimising performance are introduced to prevent ineffective searches and enhance convergence stability.

During training, samples generated by the agent’s interactions with the optical environment are stored in an experience replay pool and used to update the online policy network via random sampling in small batches; the target network is used to compute a stable target value, thereby reducing training fluctuations caused by the correlation between neighbouring samples and rapid parameter changes. Once the stopping criterion is satisfied, the candidate structure yielding the highest cumulative reward under the prescribed thickness constraints is selected, and its normal-incidence spectrum is subsequently verified through independent FDTD recalculation [38].

## 3. Results and Discussion

### 3.1. Optimisation Results

**Figure 3:**
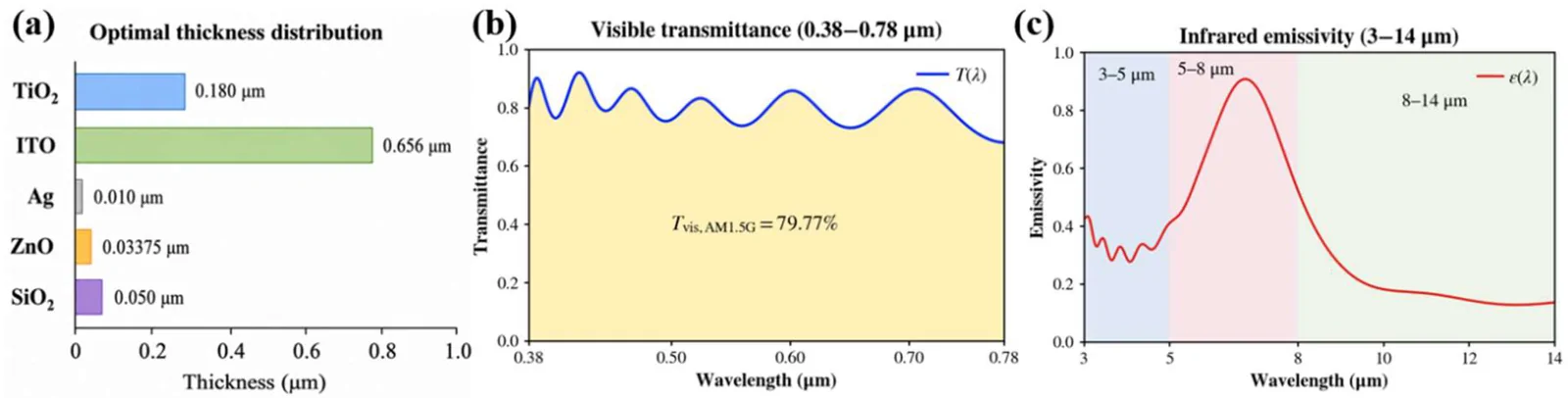
(**a**) Optimal thickness distribution of the five-layer multilayer film; (**b**) visible-light transmission spectrum; (**c**) infrared emission spectrum in the 3–14 μm range.

Based on the multi-objective reinforcement learning optimisation framework proposed earlier, this study carried out structural optimisation and feedback-based iterative validation of a transparent, infrared-selective stealth multilayer film. Figure 3a shows the optimal film thickness distribution that ultimately satisfies the comprehensive performance requirements.

The optimised transparent infrared stealth film consists of a five-layer TiO_2_/ITO/Ag/ZnO/SiO_2_ structure, with corresponding layer thicknesses of 180, 656, 10, 33.75, and 50 nm, respectively. The quantitative selection criteria and comparison of representative candidate structures are provided in Table S1 of the Supplementary Materials.

The normal spectral response of the multilayer film was obtained through FDTD full-wave electromagnetic simulation. Based on the optimisation results, a systematic analysis was subsequently conducted on the structure’s visible-light transparency, infrared-selective emission characteristics and the underlying mechanisms of its electromagnetic response.

### 3.2. Spectral Performance

Figure 3b,c show the visible-light transmission spectrum and infrared emission spectrum of the optimised multilayer film, respectively. This structure maintains a high transmittance in the 0.38–0.78 μm range, while exhibiting distinct selective emission characteristics in the 3–14 μm infrared band. Notably, the emissivity in the 5–8 μm band is significantly higher than that in the two infrared detection windows, indicating that the structure is capable of enhancing thermal radiation within the non-detection windows while suppressing mid- and long-wave infrared radiation output.

To account for the actual spectral-energy distributions, the AM1.5G standard solar spectrum and the Planck spectrum of a 300 K blackbody are used as weighting functions [39] to calculate the weighted visible transmittance and the blackbody-weighted infrared emissivity, respectively. The temperature of 300 K was adopted as a representative near-ambient reference condition, providing a consistent basis for evaluating the multiband thermal-emission characteristics of the proposed structure. The expressions are as follows:

(2) $$T_{vis}=\frac{\int_{\lambda_{1}}^{\lambda_{2}}T\left(\lambda \right)\cdot I_{AM1.5}\left(\lambda \right)d\lambda }{\int_{\lambda_{1}}^{\lambda_{2}}I_{AM1.5}\left(\lambda \right)d\lambda }$$

(3) $$\varepsilon_{band}=\frac{\int_{\lambda_{3}}^{\lambda_{4}}\varepsilon \left(\lambda \right)\cdot I_{bb}\left(\lambda ,T\right)d\lambda }{\int_{\lambda_{3}}^{\lambda_{4}}I_{bb}\left(\lambda ,T\right)d\lambda }$$

Based on weighted integration, the optimised structure exhibits a visible-light-weighted average transmittance of 79.77% and blackbody-weighted average emissivities of 33.93%, 72.93%, and 19.94% in the 3–5 μm, 5–8 μm, and 8–14 μm bands, respectively. The emissivity peaks in the 5–8 μm band, while it is markedly suppressed in the 3–5 μm band and remains low in the 8–14 μm atmospheric window. These results clearly demonstrate that the multilayer film simultaneously achieves visible transparency, selective radiative cooling, and reduced infrared emission.

### 3.3. Electromagnetic Mechanism of Spectral Selectivity

**Figure 4:**
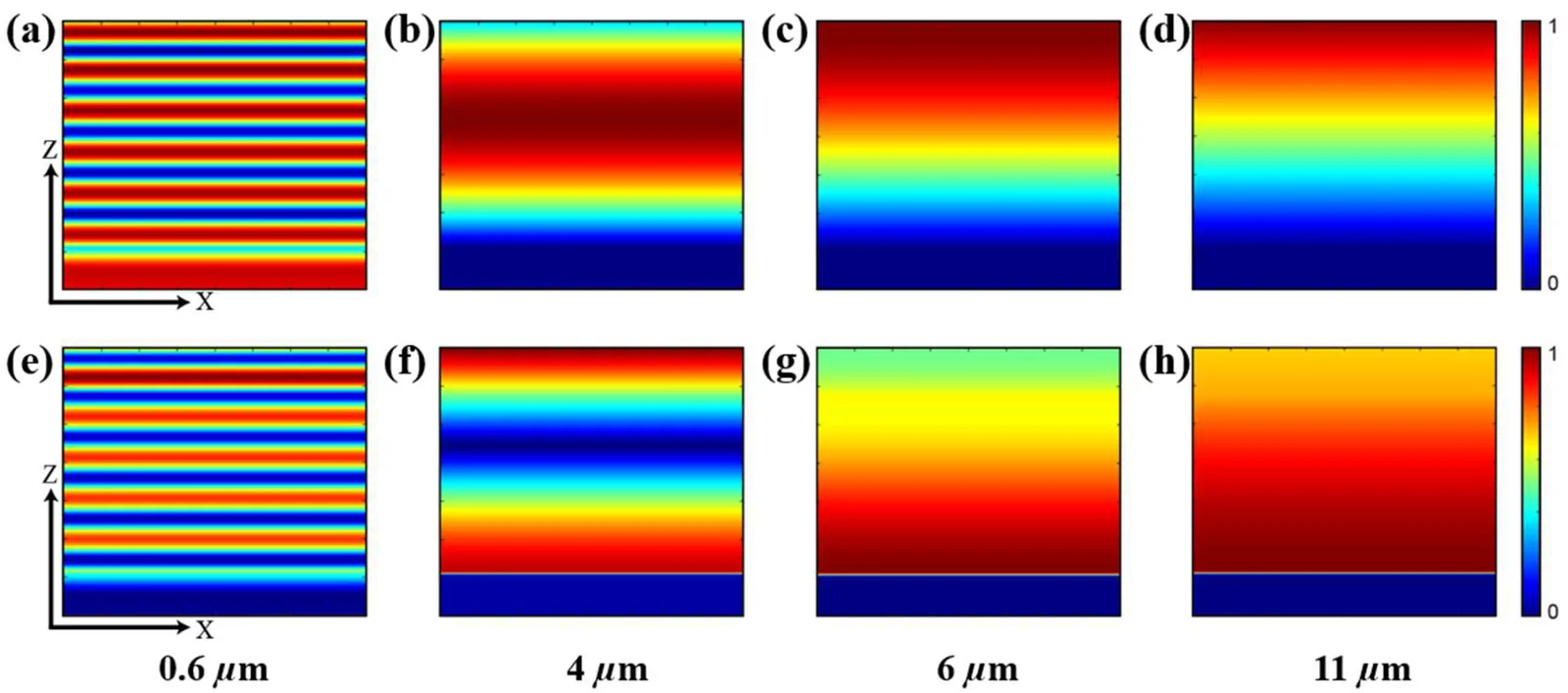
(**a**–**d**) Electric field distributions in the XOZ plane at wavelengths λ = 0.6, 4, 6 and 11 μm; (**e**–**h**) magnetic field distributions in the XOZ plane at the corresponding wavelengths.

To elucidate the physical mechanisms underlying the high visible-light transmittance, suppression of emission in the infrared detection window, and selective heat dissipation in the 5–8 μm range achieved by the designed TiO_2_/ITO/Ag/ZnO/SiO_2_ multilayer film, this paper analyses the normalised electric and magnetic field distributions within the XOZ cross-section at four typical wavelengths (0.6, 4, 6 and 11 μm), as shown in Figure 4.

Figure 4a–d and Figure 4e–h show the electric and magnetic field distributions at the four wavelengths, respectively. Comparison of the propagation, attenuation and interface localisation characteristics of the electromagnetic fields across different wavelength bands allows the formation mechanism of the multilayer film’s differentiated spectral response to be further elucidated [40,41,42,43].

#### 3.3.1. High Visible-Light Transmittance

As shown in Figure 4a,e, at a visible-light wavelength of 0.6 μm, both the electric and magnetic fields exhibit continuous, periodic striped distributions within the multilayer film, indicating that incident light is able to propagate between the individual layers and form distinct interference responses. The field distributions do not decay rapidly in the surface region but instead extend continuously into the interior of the structure, indicating that good phase matching has been achieved between the TiO_2_, ITO, ZnO, and SiO_2_ layers. The ultrathin Ag layer exerts only a limited hindrance to the propagation of visible light, and the multilayer interference further reduces interfacial reflection, consistent with the high-transmission spectrum shown in Figure 3b.

#### 3.3.2. Selective Infrared Emission

At 4 μm and 11 μm, as shown in Figure 4b,d,f,h, the electromagnetic field gradually attenuates from the incident side towards the interior of the structure, and the field strength is lower after entering the lower functional layer. Taking into account the reflection, transmission, and absorption components at the corresponding wavelengths, it is evident that incident infrared waves struggle to couple fully into the entire multilayer film; the ultrathin Ag layer and the dielectric layer jointly enhance interfacial reflection, thereby suppressing energy dissipation and radiative output within the 3–5 μm and 8–14 μm detection windows.

**Figure 5:**
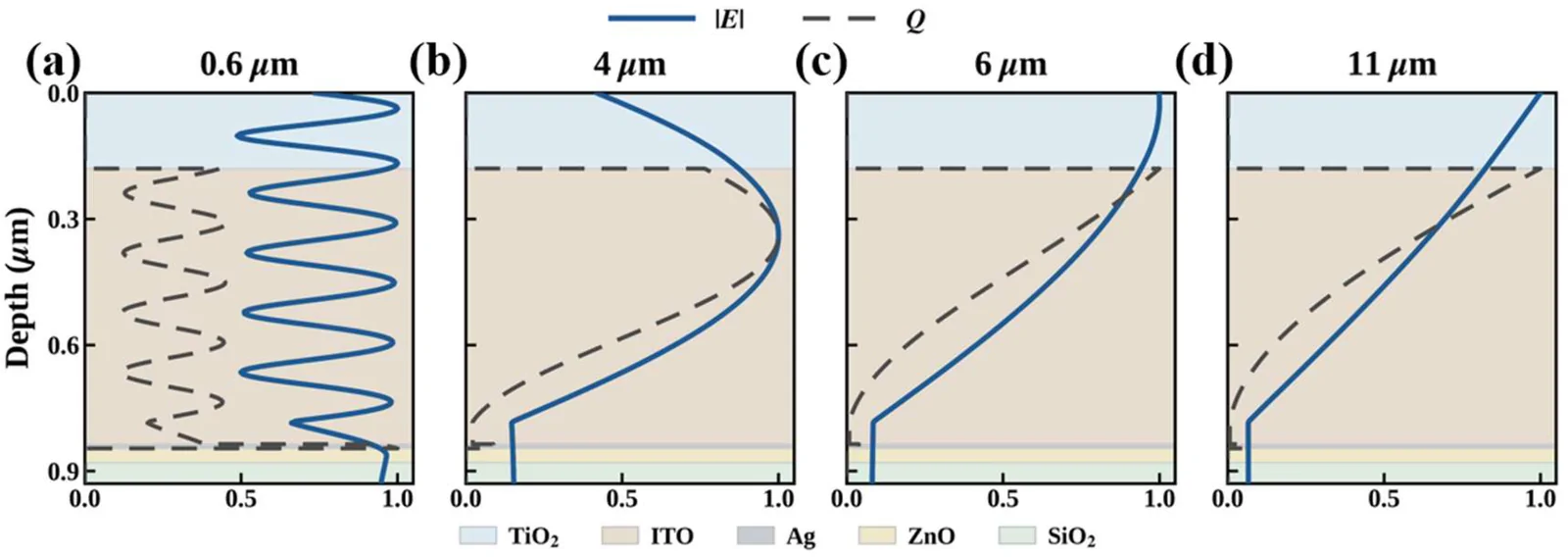
(**a**–**d**) Distribution of the normalised electric field amplitude |E| and relative loss Q along the depth of the multilayer film at wavelengths λ = 0.6, 4, 6 and 11 μm.

By contrast, at 6 μm, the electric and magnetic fields shown in Figure 4c,g extend more fully into the multilayer film and exhibit stronger spatial overlap near the TiO_2_/ITO interface and within the ITO-containing region. Combined with the higher total absorptance in the 5–8 μm band and the depth-dependent loss distribution shown in Figure 5c, this field distribution is consistent with more effective energy penetration and dissipation within the lossy functional region, thereby contributing to the elevated emissivity in this wavelength range. It should be noted that the normalised electromagnetic fields are used solely to determine the coupling locations; the actual absorption intensity also depends on material loss and the total absorption coefficient. The above results demonstrate that the multilayer film achieves selective control of emission suppression in the infrared detection window and high emission in the non-detection window through wavelength-dependent interference, reflection, and loss-coupling mechanisms.

### 3.4. Depth-Dependent Electric-Field and Loss Distributions

To further elucidate the locations of energy transfer and dissipation within the multilayer film at different wavelengths, this paper analyses the distribution characteristics of the normalised electric field amplitude |E| and the relative loss figure Q along the depth of the structure at 0.6, 4, 6 and 11 μm, as shown in Figure 5. The background colours correspond to the TiO_2_, ITO, Ag, ZnO, and SiO_2_ functional layers, respectively. The relative loss Q is obtained by normalising the localised volumetric power dissipation density after averaging it in the transverse direction for each wavelength; therefore, Figure 5 is primarily used to determine the location of loss, rather than to directly compare the absolute magnitude of loss at different wavelengths. As shown in Figure 5a, at 0.6 μm, the electric field exhibits distinct periodic oscillations within the TiO_2_ and ITO layers and extends all the way to the bottom of the structure, indicating that visible light propagates via stable interference within the multilayer film without undergoing rapid attenuation; the corresponding Q distribution primarily reflects the locations of local losses within the ITO layer. As shown in Figure 5b,d, at wavelengths of 4 and 11 μm, the electric-field amplitude gradually decreases with increasing depth. After the field reaches the Ag, ZnO, and SiO_2_ layers, its magnitude remains relatively low, while the loss is concentrated mainly in the ITO region near the incident side. Together with the low total absorptance shown in Figure 3c, these results indicate that the low emissivity in the two detection windows is governed mainly by strong interfacial reflection and limited localised dissipation.

By contrast, the electric field at 6 μm shown in Figure 5c remains relatively strong near the TiO_2_/ITO interface and gradually attenuates within the ITO-containing region, while the corresponding relative-loss distribution spans a broader effective depth. Together with the high total absorptance at this wavelength, these features support increased electromagnetic-energy dissipation within the lossy functional region and provide a physical explanation for the enhanced 5–8 μm emissivity. The wavelength-specific normalisation of the loss profiles further highlights the spatial distribution and effective depth of electromagnetic energy dissipation within the multilayer structure.

### 3.5. Polarisation- and Angle-Dependent Infrared Response

**Figure 6:**
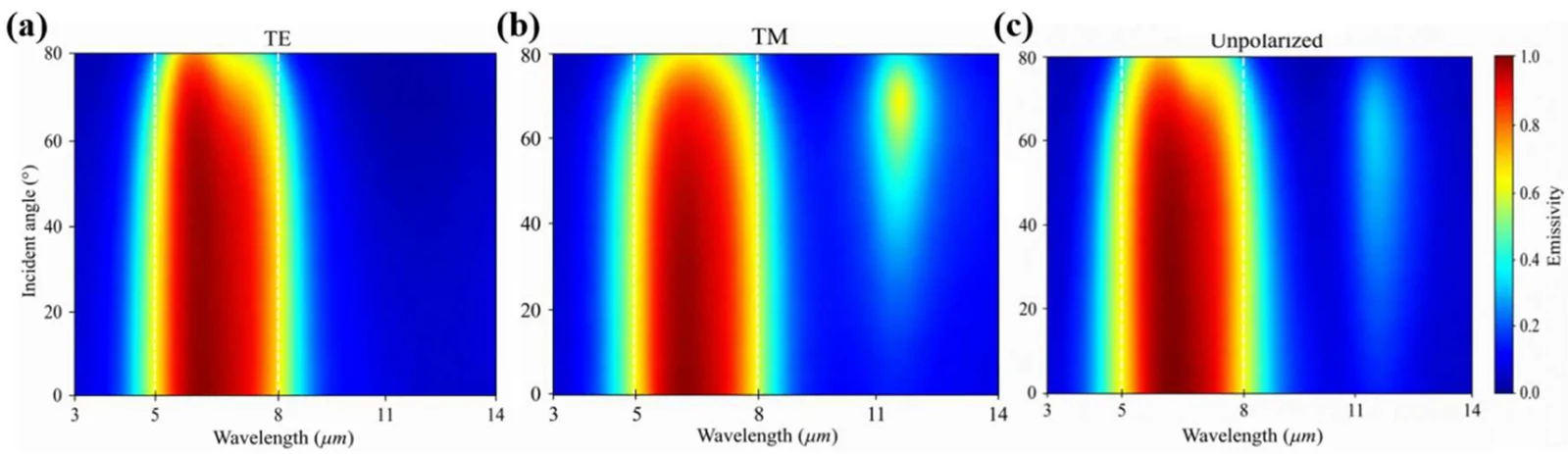
Angle-resolved emissivity distributions under (**a**) TE-polarised, (**b**) TM-polarised, and (**c**) unpolarised incidence in the 3–14 μm range, calibrated against the normal-incidence FDTD spectrum.

To evaluate the infrared spectral stability of multilayer films under different incident conditions, this paper calculated the TE, TM, and unpolarised emissivity distributions in the 3–14 μm range and for incident angles of 0–80° using the transfer-matrix method. Figure 6a–c correspond to TE-polarised, TM-polarised, and unpolarised incident conditions, respectively, with the white dotted lines marking the boundaries of the 5 μm and 8 μm bands.

As shown in Figure 6a, under TE polarisation, the high-emission band at 5–8 μm remains continuous over a wide range of angles, indicating that the selective emission characteristics in this band exhibit good angular stability; at the same time, the 3–5 μm and 8–14 μm regions generally maintain low emission levels, with only a certain degree of spectral broadening occurring at large angles. As shown in Figure 6b, the high-emission region at 5–8 μm is similarly maintained under TM polarisation; however, as the incident angle increases, additional emission enhancement gradually appears in the 8–14 μm band, being particularly pronounced at approximately 11–12 μm and in the high-angle region, indicating that the TM mode is more sensitive to oblique incidence.

As shown in Figure 6c, the unpolarised results combine both TE and TM responses; the overall distribution retains the key characteristics of high emissivity in the 5–8 μm range and low emissivity in the 3–5 μm and 8–14 μm ranges. Compared with the TE polarisation, under unpolarised conditions, the long-wave infrared region exhibits a certain degree of increased emissivity at large incident angles, whilst maintaining good spectral selectivity in the low-to-medium angle range. Overall, this structure is capable of maintaining stable radiative cooling performance in the 5–8 μm range across a wide range of incident angles, whilst maintaining relatively low emission levels within the two infrared detection windows, demonstrating a degree of polarisation adaptability and wide-angle robustness.

## 4. Conclusions

In this study, we employ a reinforcement learning-based multi-objective optimisation framework to design a transparent infrared-selective stealth multilayer film, yielding a five-layer structure of TiO_2_ (180 nm)/ITO (656 nm)/Ag (10 nm)/ZnO (33.75 nm)/SiO_2_ (50 nm). Simulation results indicate that the structure attains a weighted average visible transmittance of 79.77% over 0.38–0.78 μm. The blackbody-weighted average emissivities in the 3–5, 5–8, and 8–14 μm bands are 33.93%, 72.93%, and 19.94%, respectively, demonstrating a selective spectral profile with high visible transparency, suppressed emission in the infrared atmospheric windows (3–5 and 8–14 μm), and enhanced emission in the non-detection window (5–8 μm). To elucidate the underlying mechanisms of this differential spectral control, we further analyse the electromagnetic field distributions and depth-dependent losses. The results reveal that stable interference propagation in the visible range, field attenuation associated with interfacial reflection in the infrared windows, and enhanced field penetration and distributed energy dissipation within the ITO-containing region in the 5–8 μm band collectively underpin the film’s transparent stealth and selective thermal-radiation characteristics. After spectral calibration via FDTD, we also examine the angular response in the infrared band. The structure maintains its primary selective emission characteristics for TE, TM, and unpolarised incidence over angles up to 80°, with only a slight increase in long-wave infrared emission at the largest angle, confirming good spectral robustness. Given its multiband synergistic control and structural simplicity, this multilayer film holds significant promise for infrared protection and related applications.

## Data Availability

The original contributions presented in this study are included in the article/Supplementary Material. Further inquiries can be directed to the corresponding author.

## Supplementary Materials

The following supporting information can be downloaded at https://www.mdpi.com/article/10.3390/nano16161038/s1, Table S1: Quantitative comparison of representative multilayer configurations obtained during the optimisation search.
