# Supplementary material for: Multilayer Composite Structured Transparent Infrared-Selective Stealth Films with Synergistic Radiative Cooling
Source: Nanomaterials (Basel). 2026 Aug 20;16(16):1038. doi: 10.3390/nano16161038 (PMC13516320; doi:10.3390/nano16161038)
Supplement: Supplementary file 1 [file nanomaterials-16-01038-s001.zip › nanomaterials-4483820-supplementary.pdf]

Supplementary Materials

Multilayer Composite Structured Transparent Infrared-Selective Stealth Films with Synergistic Radiative Cooling

Juantao Zhang[1], Haining Ji[1,2*], Shisong Jin[1,2], Zhiwen Wu[1,2], Yuzhuo Ma[1,2], Jianfeng Li[1], Guanhong Lu[1], Chang Cheng[1], and Xiangle Li[1]

[1] School of Physics and Optoelectronics, Xiangtan University, Xiangtan, 411105, P. R. China

[2] Hunan Engineering Laboratory for Microelectronics, Optoelectronics and System on a Chip, Xiangtan University, Xiangtan, 411105, P. R. China

sdytjhn@126.com

S1. Quantitative selection of the optimal multilayer configuration

To clarify the quantitative basis for selecting the final multilayer configuration, three representative structures were selected from the optimisation search results. These configurations cover different material combinations, layer numbers, stacking sequences, and thickness distributions. Their AM1.5G-weighted visible transmittance, 300 K blackbody-weighted infrared emissivities, and multi-objective reward scores are compared in Table S1.

The multi-objective reward score R was calculated as

$$R = 2.6T_{vis} + 3.2\varepsilon_{5-8} - 1.5\varepsilon_{3-5} - 2.5\varepsilon_{8-14}$$
$$+0.8(\varepsilon_{5-8} - \varepsilon_{8-14}) + 0.5(\varepsilon_{5-8} - \varepsilon_{3-5}) - P, \quad \text{(S1)}$$

where T_{vis} is the AM1.5G-weighted average transmittance over 0.38-0.78 μm, and ε_{3-5}, ε_{3-5}, and ε_{8-14} are the 300 K blackbody-weighted average emissivities in the corresponding infrared bands. The soft-constraint penalty P was defined as

$$P = 8max(0,0.62 - T_{vis})^2 + 5max(0,0.72 - \varepsilon_{5-8})^2$$
$$+5max(0, \varepsilon_{3-5} - 0.32)^2 + 7max(0, \varepsilon_{8-14} - 0.24)^2$$
$$+15max(0,0.50 - T_{vis})^2. \quad \text{(S2)}$$

This criterion assigns positive contributions to visible transmittance and emissivity in the 5-8 μm radiative-cooling band. The emissivities in the 3-5 and 8-14 μm atmospheric windows contribute to the penalty terms. The contrast terms further favour selective emission in the 5-8 μm band.

The representative configurations are listed below. All thicknesses are in nm, and the sequences are given from the incident side to the substrate side.

Candidate A (nm): Al_2O_3(157)/ITO(646)/Ag(8)/TiO_2(229).

Candidate B (nm): TiO_2(248)/ITO(297)/AZO(440)/Ag(7.5)/SiO_2(91).

Candidate C (nm): SiO_2(81)/ZnO(111)/ITO(692)/Ag(7)/Si_3N_4(166)/Al_2O_3(37).

Selected structure (nm): TiO_2(180)/ITO(656)/Ag(10)/ZnO(33.75)/SiO_2(50).

Candidate A obtained ε_{5-8}=79.08% and ε_{8-14}=11.78%, while its T_{vis} was 61.02% and ε_{3-5} was 38.83%, giving a reward score of 3.956. Candidate B yielded T_{vis}=72.89%, ε_{5-8}=80.26%, and ε_{8-14}=27.94%, with a reward score of 3.988.

Candidate C achieved the highest ε_{5-8} of 84.71%, together with T_{vis}=69.06% and ε_{8-14}=27.22%, giving a reward score of 3.967.

The selected TiO_2/ITO/Ag/ZnO/SiO_2 structure achieved T_{vis} =79.77% and $\varepsilon_{3-5}/\varepsilon_{5-8}/\varepsilon_{8-14}$ values of 33.93%/72.93%/19.94%. Its reward score of 4.017 was the highest in Table S1, demonstrating the best overall balance among visible transparency, atmospheric-window emission suppression, and selective thermal emission in the 5-8 μm band.

Table S1. Quantitative comparison of representative multilayer configurations obtained during the optimisation search.

Case	T_{vis}(%)	ε_{3-5}(%)	ε_{5-8}(%)	ε_{8-14}(%)	Score
A	61.02	38.83	79.08	11.78	3.956
B	72.89	29.32	80.26	27.94	3.988
C	69.06	36.31	84.71	27.22	3.967
**Selected**	**79.77**	**33.93**	**72.93**	**19.94**	**4.017**

Note: T_{vis} is AM1.5G-weighted over 0.38-0.78 μm; the infrared emissivities are weighted using a 300 K blackbody spectrum. Decimal values were used for the reward calculation.
